# Systematic review and meta-analysis of molecular tumor board data on clinical effectiveness and evaluation gaps

**DOI:** 10.1038/s41698-025-00865-1

**Published:** 2025-04-02

**Authors:** Beryl Primrose Gladstone, Janina Beha, Arisa Hakariya, Pavlos Missios, Nisar P. Malek, Michael Bitzer

**Affiliations:** 1 Department of Internal Medicine I, Eberhard-Karls University Tübingen, Tübingen, Germany; 2 DZIF-Clinical research unit, Infectious diseases, Internal Medicine I, Eberhard-Karls University, Tübingen, Germany; 3 Center for Personalized Medicine, Eberhard-Karls University, Tübingen, Germany; 4 M3-Research Center for Malignome, Metabolome and Microbiome, Eberhard-Karls University, Tuebingen, Germany

**Keywords:** Targeted therapies, Outcomes research

## Abstract

Molecular Tumor Boards (MTBs) are pivotal in personalized cancer care. This systematic review and meta-analysis included 34 studies out of 576 articles (2020–January 2024) involving 12,176 patients across 26 major cancer entities. Of these, 20.8% (2,532 patients) received MTB-recommended therapies, with 178 outcome measures reported, achieving a median overall survival (OS) of 13.5 months, progression-free survival (PFS) of 4.5 months, and an objective response rate (ORR) of 5–57%. A pooled PFS2/PFS1 ratio ≥ 1.3 from 14 reports was observed in 38% (33–44%) of cases. Comparative data showed improved outcomes for MTB-treated patients, with hazard ratios of 0.46 (0.28–0.76, *p* < 0.001) for OS in 19 and 0.65 (0.52–0.80, *p* < 0.001) for PFS in 3 studies. These results highlight the benefits of MTB evaluations in improving outcomes for patients with solid tumors but also emphasize the need for standardized evaluation criteria to enable robust comparisons across studies.

## Introduction

In recent decades, advances in cancer research have illuminated the genetic foundations driving its initiation and progression. This understanding has paved the way for groundbreaking advancements in cancer treatment and the emergence of personalized medicine. With the invention of multi-omics approaches, major advances in cancer genomic analysis and molecular profiling have been accomplished, expanding the range of available targeted therapies for cancer patients, especially those who have exhausted their conventional treatment options^1–4^. Personalized medicine in oncology requires multiple processes. This includes identifying particular biomarkers or alterations with next-generation sequencing (NGS), searching vast databases or literature, and discussing appropriate drugs or drug combinations for each patient. These complex procedures stimulated the widespread establishment of interdisciplinary molecular tumor boards (MTB).

The MTB team broadly reviews each patient’s unique characteristics, complex molecular profiling, pathology, imaging, and clinical history to identify targeted therapies by matching the drugs to the molecular alterations or biomarkers detected, resulting in recommendations for molecular-guided personalized cancer treatments^2^. Though complex and time-consuming, an MTB referral allows cancer patients and their attending physicians to receive molecular cancer treatments outside established therapies based on the latest scientific evidence, for example, Bitzer et al.^5^, Hoefflin et al.^6^, and Luchini et al.^7^. MTB recommendations typically include the suggestions of clinical studies, in-label, off-label, or matched experimental treatments.

Clinical networks in precision oncology allow a constant improvement of these complex procedures by sharing expertize and accelerating the process so that individual hospitals may benefit from expert-agreed, consistent decision-making and structured data capture^8–12^. Despite establishing all the complex procedures along with its need for vast human and technological resources, there is no consensus on standardized, structured assessments of benefits of MTB recommendations and their assumed improvement over time. A systematic review of clinical outcomes of MTBs by Larson et al.^13^ identified 14 studies done until early 2020, pointing out the need for better quality data and recommended standardization of approaches and outcomes. The review focused on clinical outcome measures with partial, complete, and overall response rates among patients referred to MTBs but did not try to quantify the overall effect of the recommended treatments on the patients.

An essential prerequisite to accessing reliable outcome data of MTBs is to ensure the capture of high-quality, real-world data of the course of treated patients^9^, which is not readily available in most published reports describing MTB procedures and diagnostic results. With this background, we performed a systematic review and meta-analysis with the primary objective of assessing the effectiveness of MTB recommendations for cancer treatment strategies in terms of improvement of clinical outcomes among cancer patients. The secondary objectives were to describe all outcome measures reported and to identify gaps in the assessment of the effectiveness of MTBs.

## Results

### Study characteristics

**Fig. 1:**
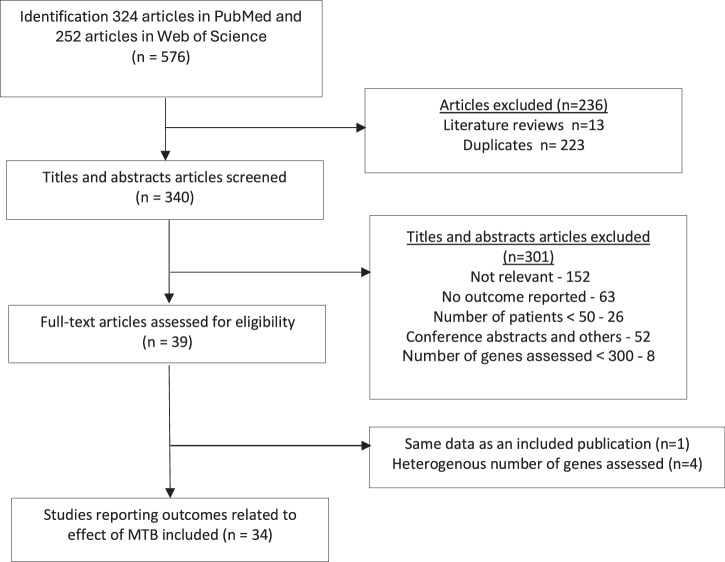
PRISMA flowchart for the search results and selection process of the included studies.

The search identified 576 articles and 340 articles were screened after removing duplicates to identify 34 MTB studies. Primary data on patient outcomes for 12.176 patients referred to the MTB and 2.532 patients (20.8%) who received MTB-recommended therapy (Fig. 1). More than half of the studies were retrospective cohort or register based studies (18/34, 52.9%), while the others were either data from clinical trials or prospective cohorts. The majority of studies (12, 35%) were from Germany followed by France (6, 18%) and USA (7, 21%). 28 studies (82%) collected their data for a period of at least 3 years. The main characteristics of the included studies are presented in Table 1 and Supplementary Table 1. Patients with any advanced cancer were included in 21 (62%) studies, while the others focused on specific cancer types (breast/gynecological cancer, non-small cell lung cancer (NSCLC), specific gastrointestinal and nervous system related cancers) and involved between 69 and 1.772 cancer patients. Overall, there were 26 major tumor entities mentioned (Supplementary Fig. 1), the most frequent being breast (1.516, 14%), lung (1.273, 12%), upper gastro-intestinal (GI) (928, 9%), lower GI (900, 8%), bone & soft tissue (876, 8%), biliary tract (691, 6%), gynecologic (658, 6%), urinary tract and kidney (559, 5%), pancreatic (511, 5%), neuroendocrine (468, 4%), and brain (462, 4%) cancers.

**Table 1:** List of included studies reporting the clinical outcomes among cancer patients treated in accordance with the recommended treatment strategy of the molecular tumor board (MTB) (*N* = 34)

No.	Reference	Country	Year of Publication	Tumor Types^a^	Age(range), Years	Number of patients referred to MTB	Number of patients treated according to MTB	Outcomes
1	Bertucci et al.^23^	France	2021	Advanced solid cancer, refractory cancer	59 (20–84)	441	94	median PFS2/PFS1 ratio pPFSR_≥1.3_ median PFS2 the 6-month PFS2 PRR CRR median PFS2 if DC 6-month PFS2 if DC median PFS2 if PD 6-month PFS2 if PD median OS 6-month OS
2	Bitzer and Ostermann et al.^5^	Germany	2020	GI tumors	-	96	25	median PFS median OS
3	Gambardella et al.^20^	Spain	2021	Advanced solid tumor	61 (24–83)	231	24	median PFS pPFSR_≥1.3_
4	Hlevnjak, Schulze, and Elgaafary et al.^44^	Germany	2021	Metastatic breast cancer (mBC)	53 (27–79)	127	64	pPFSR_≥1.3_
5	Hoefflin et al.^6^	Germany	2021	Advanced cancer	54 (1–88)	488	76	pPFSR_≥1.3_ ORR DCR OS
6	Horak, Heining, Kreutzfeldt, and Hutter et al.^45^	Germany	2021	Rare cancer	45 (16–82)	1484	362	ORR DCR pPFSR_≥1.3_
7	Huang et al.^30^	USA	2021	Newly diagnosed with non–small-cell lung cancer (NSCLC), invasive NSCLC	-	77^b^	77	OS
8	Kato and Kim et al.^31^	USA	2020	Diverse malignancy, advanced/metastatic disease	61 (3–92)	759	265	PFS OS
9	Koopman et al.^46^	Netherlands	2020	Non–small-cell lung cancer (NSCLC) with rare or complex mutational profiles	-	106	59	ORR median PFS median OS
10	Lamping and Benary et al.^32^	Germany	2020	All malignancies	51 (21–86)	70	39	median PFS median OS
11	Louie and Kato et al.^33^	USA	2022	Advanced colorectal cancer	-	87	34	PFS OS CBR
12	Louie and Kato et al.^34^	USA	2022	Pan-cancer,advanced, refractory setting	-	80^§^	80	PFS OS CBR
13	Martin-Romano et al.^47^	France	2022	Advanced cancer	59 (23–90)	516	136	median PFS median OS
14	Miller and Hutchcraft et al.^48^	USA	2022	Advanced cancer	-	679	93	median PFS pPFSR_≥1.3_
15	Pleasance and Bohm et al.^49^	Canada	2022	Advanced or metastatic cancer of diverse types	57 (19–86)	570	209	median PFS
16	Reda et al.^22^	France	2020	A locally advanced non-operable or metastatic cancer	-	386	79	median PFS2
17	Sultova et al.^50^	Germany	2020	Metastatic breast cancer or gyneco-logical malignancies	52 (19–82)	95	9	pPFSR_≥1.3_
18	Sultova et al.^51^	Germany	2021	Metastatic breast cancer	52 (30–82)	100	16	pPFSR_≥1.3_
19	Tarawneh et al.^38^	Germany	2022	Advanced-stage malignancies, solid malignancies	57.5 (22-77)	104	33	PFS
20	Cobain and Wu et al.^52^	USA	2021	Advanced solid tumor	57,7	1015	132	best response rate, exceptional responder rate
21	Bayle and Belcaid et al.^53^	France	2023	Metastatic solid tumors	63 (54–71)	1772	122	median PFS median OS
22	Boilève et al.^25^	France	2023	Neuroendocrine neoplasm (NEN)	55 (41–64)	114	19	
23	Zhang et al.^54^	Germany	2023	Biliary tract cancer (BTC)	62 (24–81)	153	14	median PFS pPFSR_≥1.3_ ORR median DoR median OS
24	Giacomini et al.^29^	Italy	2023	Any neoplastic condition	-	124	22	OS PFS pPFSR_≥1.3_
25	Repetto and Crimini et al.^21^	Italy	2023	Patients with tumors	54 (19–85)	251	76	ORR median PFS median OS pPFSR_≥1.3_ DCR
26	Helali et al.^27^	China	2023	Advanced solid tumors, treatment-refractory solid cancers	60 (11–95)	122	77	median OS ORR DCR
27	Debien et al.^26^	France	2023	Metastatic rare cancers, refractory rare cancers	51 (19–84)	252	49	median PFS pPFSR_≥1.3_ median OS
28	Ladekarl et al.^55^	Denmark	2023	Late-stage cancer, incurable, progressing, and/or life-threatening cancer	-	163	16	CBR ORR
29	Renovanz, Kurz, and Rieger et al.^56^	Germany	2023	Advanced tumors in the nervous system	-	408	86	pPFSR_≥1.3_ DCR median DoR median PFS
30	Pinet and Durand et al.^36^	France	2023	Advanced / Refractory solid tumors	-	69	13	median OS
31	Fukada et al.^28^	Japan	2023	Metastatic cancer	58 (12–85)	713	45	OS
32	Scheiter et al.^37^	Germany	2022	Advanced disease	59	251	47	CBR pPFSR_≥1.3_ median PFS DoCB
33	Blobner et al.^57^	Germany	2023	Recurrent or progressive glioma	-	73	12	Median PFS pPFSR_≥1.3_
34	Mosteiro, Azuara, Villatoro, and Alay et al.^35^	Spain	2023	Non-small-cell lung cancer	64 (35–88)	200	28	median OS

^a^As described by the study authors.

^b^Number of patients selected for a case control study.

^§^selected subset of patients whose MTB-recommended treatment regimen included immune checkpoint inhibitors.

*PFS* progression free survival, *pPFSR≥1.3* the percentage of the patients with a PFS2/PFS1 ratio ≧ 1.3, *PRR* partial response rate, *CRR* complete response rate, *DC* disease control, *OS* overall survival, *ORR* objective response rate, *DCR* disease control rate (CR + PR + SD), *CBR* clinical benefit rate, *DoR* duration of response, *DoCB* duration of clinical benefit.

Detailed information on the inclusion criteria for MTB presentation was available in 29 of the 34 studies. These criteria, which guide the use of next-generation sequencing (NGS) diagnostics and subsequent MTB evaluation, were as follows: advanced solid tumors irrespective of treatment line (13/29), lack of further established therapeutic options (10/29), rare cancers (7/29), disease progression during at least one line of prior therapy (5/29), patient age ≤50 years (2/29), relapse following initial remission (1/29), and recurrent glioma (1/29). Regarding the participants and their pivotal roles in the MTB, 21 out of the 34 studies (62%) provided information on the professional disciplines involved. Clinical and/or medical oncologists were included in all 21 studies. Other key disciplines represented were pathology (19/21), human or clinical genetics (15/21), bioinformatics and data science (13/21), molecular biology or molecular pathology (13/21), basic or translational science (9/21), radiology (5/21), clinical pharmacology (4/21), clinical trial coordination (4/21), and structural biology (1/21).

The median number of patients referred to MTB per study were 216 patients (range 69 to 1772 with IQR: 104–516; *N* = 34), of which a median of 99 (range 32–1138 with IQR: 57–255; *N* = 28) patients received treatment recommendations by MTB per study and a median of 54 (range 9–362 with IQR: 24–86; *N* = 34) patients were treated according to MTB recommendations. The median age of patients referred to MTBs ranged between 45–64 years with the lower and upper limits between 1–54 years and 64–95 years respectively. The patients were followed up at a median interval of 8 (range 6–26; *N* = 20) weeks from the start of MTB-recommended therapy, with 20 (59%) studies using the Response Evaluation Criteria in Solid Tumors (RECIST 1.1) criteria for assessment^14^. The median duration of patient follow-up was 11 months (IQR: 9–15; *N* = 10) ranging between 7 and 36 months. Individual data for all patients receiving MTB-recommended treatment was provided in 26 (77%) studies, however, only 12 (35%) provided outcome data for all patients and 9 (27%) only for a subset of patients.

19 (56%) of the 34 MTBs utilized prespecified actionability scales to classify recommendations; 14 used the ESMO Scale for Clinical Actionability of molecular Targets (ESCAT)^15^ alone or in combination with other scales, such as NCT/DKTK from the National Center for Tumor Diseases (NCT) and the German Cancer Consortium (DKTK)^16^, while 5 used various other actionability scales (Supplementary Table 1). Data on performance status was provided in 17 (50%) studies, with ECOG being the most frequently used scale (13, 38%). The turn-around time (TAT) with varying definitions of this period was reported in 13 (38%) studies. Among 9 MTB studies using similar definitions, the median TAT varied between 12–115 days.

Descriptive characteristics of included studies are presented in Supplementary Table 1.

### Outcomes among patients on MTB-recommended therapy

Overall, 186 outcome measures were reported with 42 (23%) related to PFS, 21 (11%) to OS, 23 (12%) to DCR, and 20 (11%) to ORR among 26, 22, 23 and 20 studies, respectively. All outcome measures are listed in Table 2 and Supplementary Table 2. Of note, PFS was the most frequently studied outcome investigated in 26 of the 34 studies (77%). A high variation was noticed in the required period to fulfill the criteria to define SD, ranging between 6 weeks and 6 months (Table 2). Patients with MTB-recommended therapies had a median OS of 13.5 (10.9–19.5) months and a median PFS of 4.5 (2.8–6.5) months. The ORR ranged from 5% to 57% and DCR from 29% to 84%, with a median partial response rate of 18% (range: 3%–57%) and complete response rate of 1% (range: 0%–8%). The PFS at 6 months reported in 3 studies were 2%, 28% and 79% respectively.

**Table 2:** Reported outcome measures among patients treated with MTB-recommended therapy and its details reported in the included studies (N = 34)

Outcome measures	Number of outcome measures	Range	Median (inter quartile range)
Partial response rate (%)	21	3–57	18 (13–29)
Complete response rate (%)	19	0–8	1 (0–3)
Stable disease rate (%)	21	5–63	25 (22–32)
Defined as at least 8 weeks	10	15–63	24 (22–34)
Defined as at least 3 months	3	5–31	24 (5–31)
Defined as at least 6 months	2	26–30	-
Objective response rate (%)	20	5–57	22.5 (12.5–30.5)
Disease control rate (%)	23	29–84	52 (36–55.3)
Median OS (months)^a^	20	2.2–35.1	13.5 (10.9–19.5)
Median PFS (months)^a^	22	1.8-15	4.5 (2.8–6.5)
OS at 6 months (%)	1	62	-
PFS at 6 months (%)	3	2–79	28 (2–79)
Median PFS2/PFS1 ratio (pPFSR)	3	0.91-1.71	1.45 (0.91-1.71)
PFS2/PFS1 ratio ≥ 1.3 (%) (pPFSR_≥1.3_)	14	25–67	36 (31–47)
Exceptional responder rate^b^	1	19.7	
Median duration of response (months)	1	5.5	
Median duration of clinical benefit (months)	1	4.8	
Clinical benefit based on treatment duration (%)	1	37	

^a^4 OS and 3 PFS outcome reports provided only hazard ratios from the survival models.

^b^Exceptional responders received sequence-directed therapy for a duration of 12 months or longer.

*OS* overall survival, *PFS* progression free survival.

**Fig. 2:**
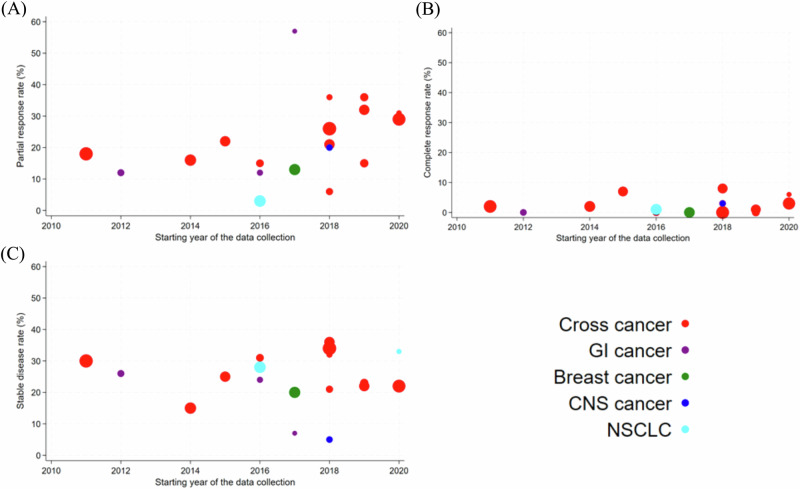
Partial response, complete response and stable disease rates among patients on MTB-recommended therapy. Red circle refers to cross cancer, purple circle refers to GI cancer, green circle refers to breast cancer, blue circle refers to CNS cancer, turquoise circle refers to NSCLC. The size of the circle represents the number of patients on MTB recommended therapy. GI Gastro-intestinal, CNS Central Nervous system, NSCLC Non-small-cell lung cancer. Panel (**A**) presents partial response rates, (**B**) presents complete response rates and (**C**) presents stable disease rates.

**Fig. 3:**
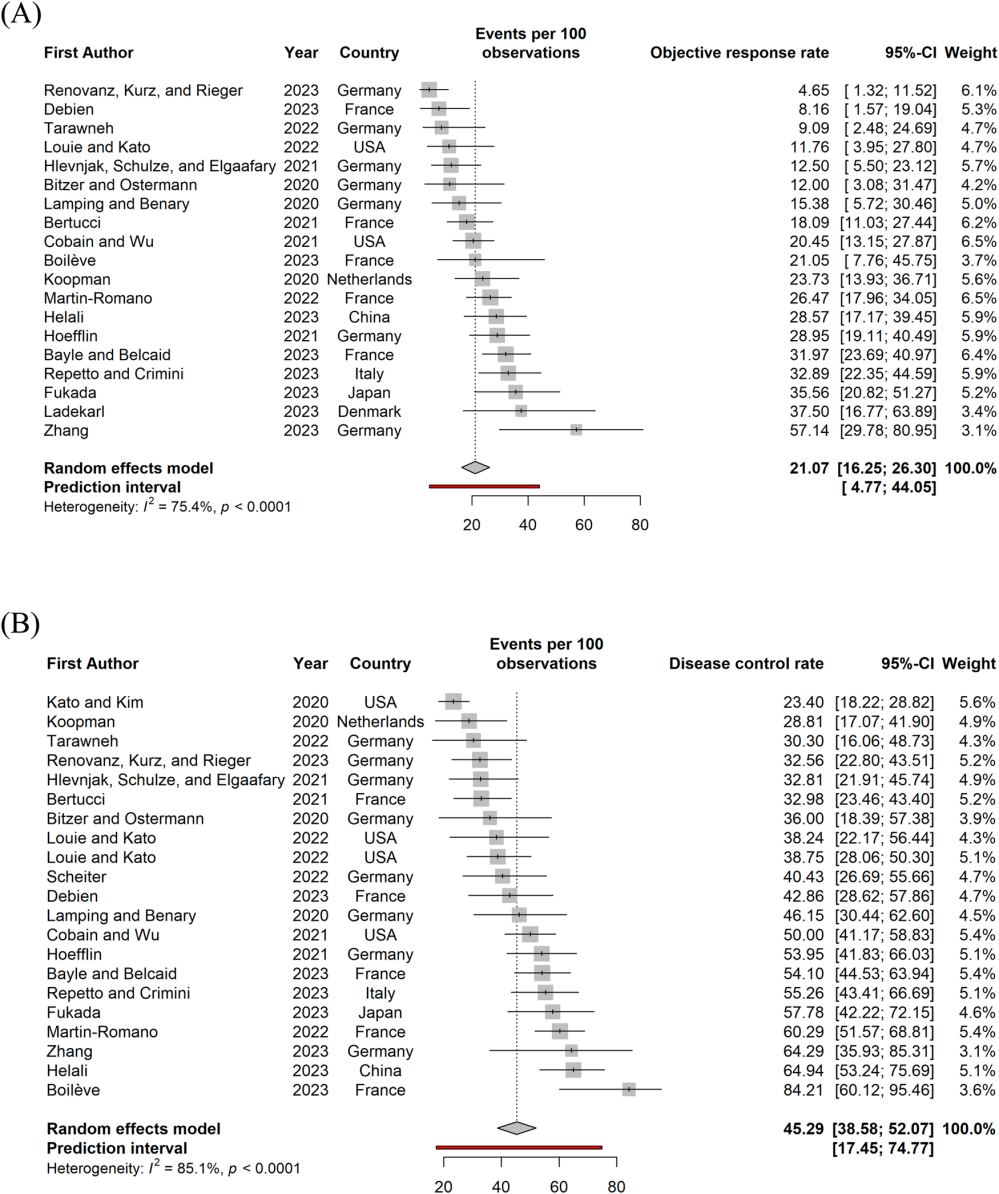
Forest plots of objective response and disease control rates among patients on MTB-recommended therapy in the included studies. Panel (**A**) presents objective response rates and (**B**) presents disease control rates. The point and the horizontal line represent the observed study estimate and its confidence interval. The size of the gray square box varies according to the weightage given to the estimate. The gray diamond represents the pooled estimate, and its length symbolizes its confidence interval. The vertical reference line indicates no effect. The red line represents the prediction interval.

**Fig. 4:**
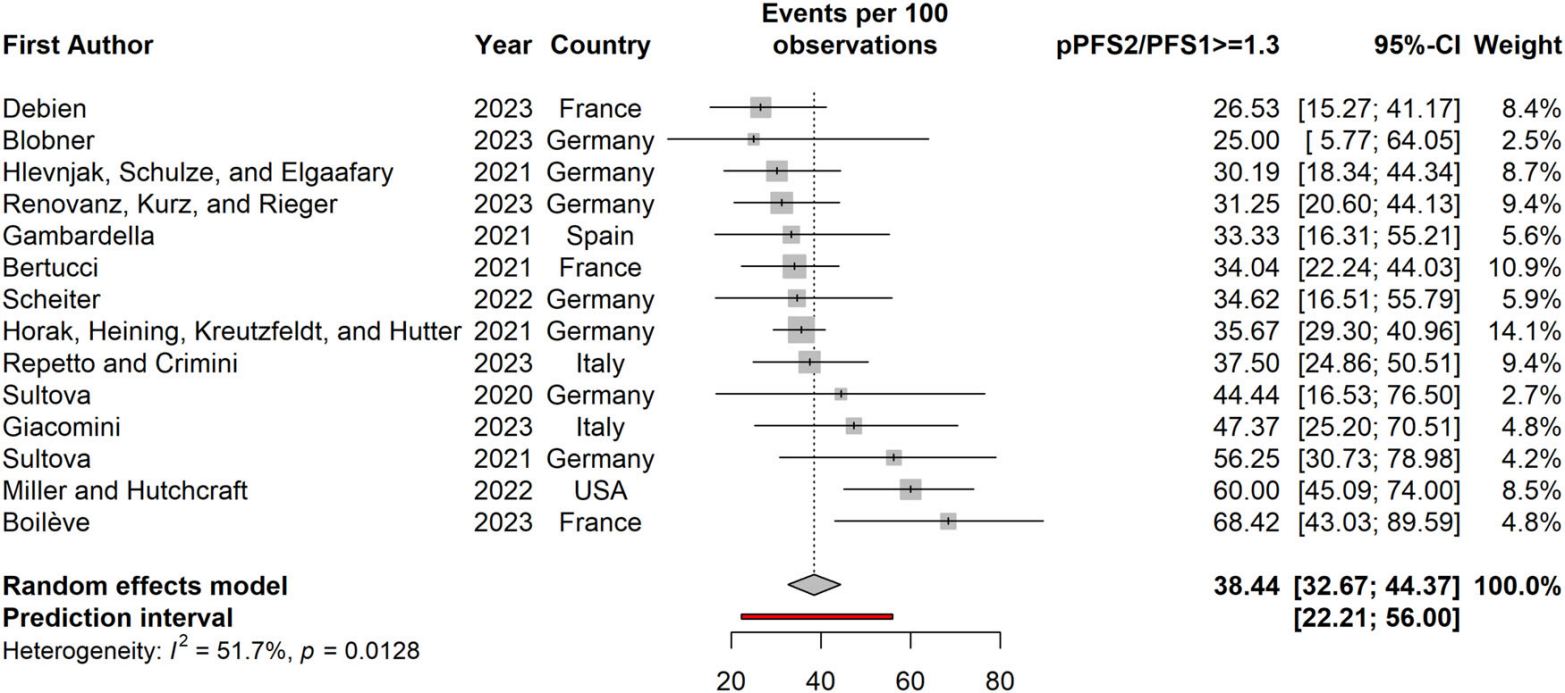
Forest plot of proportion of PFS2/PFS1 ratio greater than or equal to 1.3 among patients on MTB-recommended therapy. The point and the horizontal line represent the observed study estimate and its confidence interval. The size of the gray square box varies according to the weightage given to the estimate. The gray diamond represents the pooled estimate, and its length symbolizes its confidence interval. The vertical reference line indicates no effect. The red line represents the prediction interval.

Figure 2 shows the CR-, PR-, and SD-rates along with the respective cancer types, sample size and the starting year of the data collection. The pooled estimates of the CR-, PR-, and SD-rates were 1% (95%CI: 0%–3%), 19% (95%CI: 15%–24%), and 25% (95%CI: 21%–30%) respectively. The pooled estimates of ORR and DCR were 21% (95%CI: 16%–26%) and 45% (95%CI: 39%–52%) as shown in Fig. 3.The ratio of PFS on a MTB-recommended treatment (PFS2) to the PFS on the last previous line of therapy (PFS1) to assess whether patients benefitted from the treatment, introduced by Von Hoff et al.^17^, was reported in 14 studies. A ratio of PFS2/PFS1 ≥ 1.3 to 1.5 is considered as clinical benefit^18,19^ and proportion of patients with PFS2/PFS1 ratio ≥1.3 (pPFSR ≥ 1.3) was found to vary between 25 and 68% with a pooled estimate of 38% (33–44%) as seen in Fig. 4.

An association of predefined actionability scales to clinical outcomes was investigated in 7 (21%) studies (details are shown in Supplementary Table 3). All 5 MTBs using ESCAT or NCT/DKTK scales showed a larger benefit in survival or response rates among patients with the highest level of evidence (ESCAT tier I/II or NCT/DKTK m1A-C), a significant association of outcome parameters and evidence levels was found in 4 of these studies. MTBs applying the OncoKB therapeutic level of evidence (*N* = 1) or a so-called University of Kentucky grading of evidence scale (*N* = 1) could not establish an association of the outcomes with the actionability scale. The effect of the performance status on clinical outcomes was only studied in 3 studies. While multivariate analyses by Gambardella et al.^20^ found an independent positive association of ECOG performance status on PFS2, Repetto and Crimini et al.^21^ did not find any effect. An unadjusted positive effect of WHO status on clinical outcome was reported by Reda et al.^22^.

### Effect measures in comparison to control groups

A comparison between patients with MTB-recommended treatments and any control group was performed in 19 (56%) studies, and 3 studies had 2 comparison groups each. Overall, 5 different kinds of control groups were reported: (i) patients who were not treated in accordance with the MTB recommendations / standard of care therapies (15 studies, 44%), (ii) patients with no actionable driver / did not receive MTB recommendation (2 studies, 6%), (iii) patients who received a recommended treatment with a low study-defined matching score (2 studies, 6%), (iv) MTB referred patients who were not given any treatment (2 studies, 6%) or (v) patients not referred to the MTB (1 study, 3%). The definitions of matching scores or matched therapies varied slightly, though overlapping (details provided in Supplementary Table 4).

In total, 38 separate comparisons were reported, including 12 hazard ratios (4 PFS, 8 OS), 2 odds ratios (DCR) and 2 ratios (1 PFS, 1 OS) as effect measures. The remaining 22 comparisons did not provide any effect measure but rather assessed statistical significance using standard tests: 6 for median PFS, 3 for PFS-rate at 6 months, 7 for median OS, 1 for OS-rate at 6 months, 1 for median PFS2/PFS1ratio, 2 for pPFSR_≥1.3_, 1 for DCR, and 1 for CRR. Patients on MTB-recommended therapy numerically had a better outcome in 36/38 (95%) comparisons. Of note, 24 (66.7%) of these comparisons reached statistical significance.

**Fig. 5:**
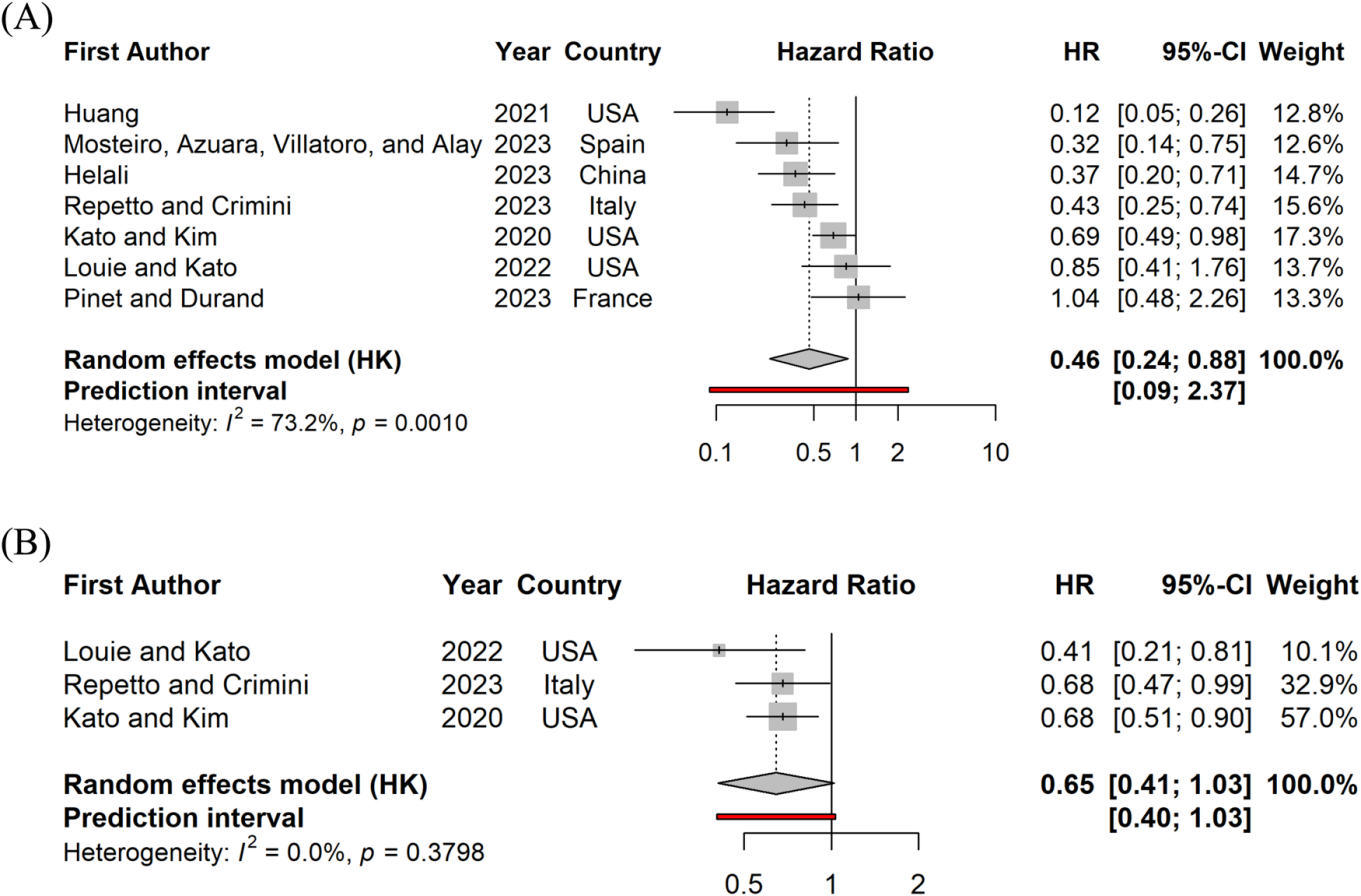
Forest plot of hazard ratios comparing overall and progression free survival among patients on MTB-recommended therapy to the comparison group. Panel (**A**) presents overall survival and (**B**) presents progression free survival. The point and the horizontal line represent the observed study estimate and its confidence interval. The size of the gray square box varies according to the weightage given to the estimate. The gray diamond represents the pooled estimate, and its length symbolizes its confidence interval. The vertical reference line indicates no effect. The red line represents the prediction interval.

Meta-analysis of the HRs for OS estimated a pooled HR of 0.46 (0.28 to 0.76, *p* < 0.001) with an I2 of 73.2% (*p* = 0.001; Fig. 5A). Similarly, patients receiving MTB recommended treatment had a significant better progression free survival (pooled HR of 0.65 (0.52 to 0.80, *p* < 0.001) with an *I*^2^ < 1% (*p* = 0.38; Fig. 5B)).

**Fig. 6:**
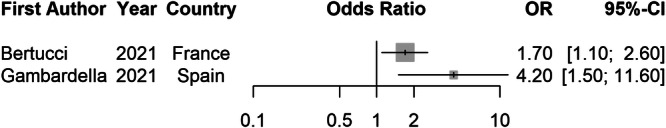
Forest plot of odds ratio of PFS2/PFS1 ratio greater than or equal to 1.3 among patients on MTB-recommended therapy as compared to the comparison group. The point and the horizontal line represent the observed study estimate and its confidence interval. The size of the gray square box varies according to the weightage given to the estimate. The vertical reference line indicates no effect.

The pPFSR_≥1.3_ was significantly higher among the MTB patients as compared to control groups with a rate ratio of 1.7 and 4.2 in 2 studies as shown in Fig. 6. There were 2 studies reporting HR of PFS among patients with advanced or metastatic malignant disease and patients with advanced colorectal cancer, both among US patients, reporting an unadjusted HR of 0.68 (0.51 to 0.90) and an adjusted HR of 0.41(0.21 to 0.81) respectively. The median PFS was more than twice longer among the MTB patients as compared to the control patients in 5 studies based in Italy, France, Spain, Germany and USA (14.2 vs. 5 months, 8.5 vs. 5.7 months, 6.5 vs. 2.8 months, 6.4 vs. 3 months, and 4.3 vs. 1.9 months), while it was similar in data from advanced French cancer patients between 2016–2018 (2.5 vs. 2.4 months). Bertucci et al compared MTB and control groups of advanced cancer patients from 2014–2019 in France according to best response assessment. That study found a higher PFS at 6 months with 79% vs. 41% among patients with disease control (CR, PR or SD), 2% vs. 1% among those with progressive disease (PD), and 28% vs. 16% overall^23^.

**Fig. 7:**
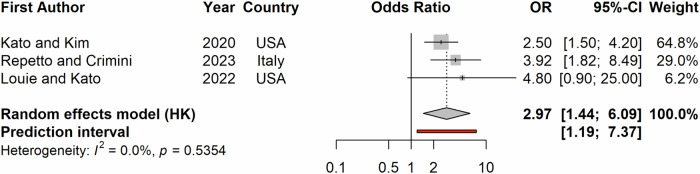
Forest plot of odds ratio of disease control rates among patients on MTB-recommended therapy as compared to the comparison group. The point and the horizontal line represent the observed study estimate and its confidence interval. The size of the gray square box varies according to the weightage given to the estimate. The gray diamond represents the pooled estimate, and its length symbolizes its confidence interval. The vertical reference line indicates no effect. The red line represents the prediction interval.

Meta-analysis for DCR showed a significant benefit among patients receiving MTB recommended treatment with an odds ratio of 2.97 (1.44–6.09), p with an *I*^2^ < 1% (*p* = 0.001; Fig. 7). Patients with diverse advanced or metastatic malignancy in the US provided an adjusted odds ratio of 0.40 (0.24 to 0.67) while comparing those with a study-defined matching score ≥50% versus <50%. Similarly, an adjusted odds ratio of 0.21 (0.04 to 1.06) for disease control of at least 6 months among US patients was reported for advanced colorectal cancer on unmatched therapy (*n* = 17) in comparison with matched therapy (*n* = 34). Further comparisons reported a significantly higher DCR among MTB patients (53% vs. 21%, *p* = 0.019) from 2012–2018 in the US with a high (≥50%) versus low (<50%) matching score and a 2% vs. 1% complete response rate among MTB patients (*p* = 0.249) with a study-defined matched versus non-matched therapy from 2014–2019 in France.

### Risk of bias assessment

Though all included studies provided relevant outcome measures of interest, the impact of MTB on patient outcomes was not the primary aim for some studies. With this background, 8 (24%) studies were assessed to be of good quality, 24 (71%) medium and 2 (6%) of low quality (Supplementary Fig. 2). Half the studies did not have a comparison group; those with comparison groups posed a high risk of bias in the area of detection of confounders, adjustment for confounding and statistical analysis. For questions regarding outcome assessment and follow-up which included outcome definition, length of follow-up, follow-up data, and incomplete follow-up, there was a low risk of bias for >70% of the studies (Supplementary Fig. 3).

## Discussion

With the widespread implementation of MTBs in routine oncology practice^2,24^, there is a high unmet need to assess patients’ clinical benefit and compare these parameters among various patient populations and periods. To date, standardized parameters to validate these assessments are yet to be proposed, and a consensus on the specificities of data, which needs to be analyzed in this context, still needs to be defined.

Our investigation encountered highly heterogeneous estimates of effect and outcome measures, reflecting the diverse cancer types, molecular alterations, treatment protocols, hospital settings, and expertize availability across the included studies. Despite these limitations, our analyses revealed remarkable results. Patients with MTB-recommended therapies had a median OS of 13.5 (10.9–19.5) months and a median PFS of 4.5 (2.8–6.5) months. From 38 separate comparisons between patients treated according to MTB recommendations and any control group, 36 had a numerically better outcome, with 67% reaching statistical significance. Importantly, our systematic review and meta-analysis, evaluating the impact of molecular tumor boards on clinical outcomes of cancer patients compared to study-defined control groups, found a significant positive effect in terms of OS, PFS, and DCR. Addressing methodological concerns regarding the definition of relevant control groups, our meta-analysis of OS data revealed a 54% lower hazard of dying compared to those not treated according to MTB recommendations. Even the intra-patient comparison of MTB-guided therapies with the previous therapy, reflected by over a 30% increase of PFS2 over PFS1 (pPFSR_≥1.3_)^18,19^, showed a significant improvement compared to non-MTB-recommended therapies.

The investigated publications reflect an effort to quantify the benefit of precision medicine within the investigated period. However, a critical finding of our systematic review is a need for enhanced standardization in endpoints and reporting of outcome measures. Important aspects to be considered in outcome selection are the type of response to MTB-guided therapies regarding sensitivity or resistance and the clinical magnitude of benefits^2^. The currently available evidence included important survival estimates such as PFS and OS, with PFS reported more often than OS. There was a high variation in the statistics reported; for example, the authors reported PFS at 6 months, median PFS, or overall PFS. Outcome measures quantifying treatment responses such as DCR and ORR among patients receiving MTB-recommended treatments could be estimated only in 68% and 59% of the studies, respectively. Though we restricted our meta-analysis to predefined parameters, we documented all the reported clinical outcome measures and picked up subtle to considerable differences in the definitions of endpoints of interest, such as criteria used to define SD varying between - at least 6 weeks, 8 weeks, 3 months, or 6 months. Such discrepancies add to the background noise of an already heterogeneous multidimensional data. Furthermore, only 62% of the studies provided patient level data on outcomes. Standardization efforts of study designs, data collation, analytical techniques, and reporting are much needed now when increasing evidence is reported through available multiple data sources and advanced data collections.

Actionability scales that define clinical evidence-based criteria to prioritize alterations, in contrast to relying solely on expert opinions, were reported in 19 of the 34 analyzed studies. However, an association between evidence levels and outcome parameters was investigated in only 7 of these studies. Notably, all studies that analyzed ESCAT or NCT/DKTK evidence levels demonstrated a greater benefit in survival or response rates among patients with the highest evidence levels.

Considering all the studies we included in this meta-analysis, excluding patients from the analysis and selecting a relevant control group pose challenges in assessing the benefit without any bias. Prospective cohort studies with appropriate comparison groups represent the minimum requirement for proper evaluation of effectiveness. Among the 34 studies included in this analysis, only 19 reported control groups for their investigations^6,20–23,25–38^. Patients in these control groups were either not treated according to the MTB recommendation, MTB referred patients with no actionable driver, not referred to the MTB, or grouped according to a predefined matching score for the MTB-guided therapy. Disparate definitions of these control groups hinder inter-study comparison and pooling of results.

Potential control groups for the study of MTB effectiveness can be chosen at different levels (Supplementary Fig. 4); however, each choice comes with different challenges. A comparison with patients not referred to MTB or patients with MTB recommendations but treated with an alternative therapy or best supportive care (BSC) may have primarily induced a bias towards a worse performance status. Moreover, the therapy line to be used to compare patients not referred to the MTB needs to be clarified; maybe the most suitable comparison group to investigate a potential benefit for MTB presentations is patients without an MTB recommendation. However, even for this comparison, a clear baseline, e.g., the date of the first new therapy regime after the MTB recommendation, has to be thoroughly defined. Additionally, in heterogeneous patient groups such as “all comers” with advanced disease, selecting the right control group becomes critical. Potential control groups could include patients with similar histology but no actionable mutation, or patients with a similar alteration but without access to the recommended treatment. Each of these options carries inherent challenges, such as biological variability between tumors or differences in treatment access, which must be accounted for during study design. Furthermore, the absence of actionable alterations seems to influence the prognosis of patients at least in some tumor entities, as e.g. recently reported by Nakamura et al.^39^. External or synthetic control arms primarily derived from real-world data to supplement the comparison in oncological research^40^ are a new development considered in precision medicine and may also be applicable to MTB research as well^41^. Most of these study designs demand using propensity scores to adjust and minimize any confounding owing to control selection processes.

Standardization of patient flow and reimbursement for molecular profiling and MTB structures, as exemplified by the Centers for Personalized Medicine in Germany^9^, is imperative. Benefit assessments from care providers’ and patients’ perspectives, including quality-of-life endpoints, are warranted. This meta-analysis advances beyond Larson et al.^13^ by providing a more detailed summary and analysis of outcome measures, an overview of MTB inclusion criteria and actionability scales, as well as an analysis of control group outcomes. Altogether, our analysis should stimulate the discussion on defining parameters that should be reported in MTB-cohort analysis, e.g., by an interdisciplinary consensus conference.

Our systematic review may be limited by the inclusion criteria focusing on the large sample size and the number of genes studied, potentially leading to homogenized results. However, having a minimum sample size and a defined study period is crucial to reflect the standard functionality of MTBs. The concentration of studies in high-income countries potentially limits generalizability; however, it reflects the historical establishment of MTBs and advancements in precision oncology research in this context.

In conclusion, our systematic review and meta-analysis based on the evidence base between 2020 and early 2024 show that MTBs positively impact the course of patients with advanced solid tumors. Notably 67% of the reported clinical outcomes from various MTB studies showed significant benefits. Importantly, this study identifies a significant positive effect of MTBs on the clinical outcomes of cancer patients, including Progression-Free Survival (PFS), Overall Survival (OS) and disease control rates (DCR). Even in comparison with previous therapies, MTB-guided treatments exhibit significant improvement in PFS2 over PFS1 (pPFSR_≥1.3_). Our findings underline the need for a structured evaluation and reporting of MTB benefits, which requires particular focus. We recommend discussing a consensus for assessing relevant parameters that should be standardized between MTB groups. This approach could tremendously improve MTB research, including comparing MTB-guided therapies between patient cohorts, regional areas, or available drug regimens.

## Methods

This review was performed following Preferred Reporting Items for Systematic Reviews and Meta-Analyses guidelines for systematic reviews and the protocol registered in PROSPERO under CRD42023404806.

### Literature search and selection process

An extensive literature search was conducted on February 1st, 2023, and later updated in October 2024 in PubMed and Web Of Science in accordance with the Cochrane Handbook of Systematic reviews using the search terms *“molecular tumor board” OR “molecular tumor board” OR “precision medicine tumor board” OR “precision medicine tumor board”* to identify all articles published between January 1st, 2020 and January 31st, 2024. Interventional or observational studies based on primary data on MTBs reporting outcomes of clinical, economic or any other impact published in English were included. Additionally, previous systematic reviews and references were combed through to identify any missed articles. Studies involving <50 patients, with molecular profiling based on <300 genes and focus on acute hematological cancer patients were excluded.

Title and abstract screening followed by full text screening were conducted by two reviewers (JB and PB) and discrepancies sorted out through discussion with MTB experts (PM, MB). The researchers were not blinded to study authors or location. Data from included articles were entered onto a standardized pre-formatted data sheet, cross-verified, and inconsistencies sorted out by consensus. Data related to publication, target population, study design, MTB-recommended therapy, follow-up, clinical outcomes and comparison of outcome measures were extracted.

### Data synthesis and meta-analysis

Primary outcomes were objective response rate (ORR), disease control rate (DCR), overall survival (OS) and progression free survival (PFS). ORR was defined as percentage of patients who achieved partial (PR) or complete response (CR) among patients receiving MTB-directed therapy. DCR was defined as percentage of patients who achieved stable disease (SD), partial response (PR), or complete response (CR) among patients receiving MTB-directed therapy. Variance estimates were calculated for the outcome measures whenever possible. Pooled estimates of outcome measures were obtained using random effects meta-analysis and heterogeneity studied using I-squared statistic when outcome or effect measures were available for 3 or more studies. Adjusted and unadjusted hazard ratios (HR) were log transformed for meta-analysis. The rates are presented as median and inter quartile range (IQR). The effect measures in the form of HR, odds ratio (OR) or rate ratio (RR) are presented as the estimate with 95% confidence intervals (CI). Heterogeneity was assessed using I-squared (I2). One study was excluded from the analysis of effect measures in comparison to the control group since the comparison group included patients on existing standard therapies, which was excluded for the MTB cohort^37^. Quality of eligible studies were assessed using Joanna Briggs quality assessment criteria (JBI)^42^ adapted to studies reporting outcomes from MTB-based data. Ethical approval was not required as all data are based on published studies. All statistical analyses were carried out using Stata version 15.1^43^.

## Supplementary information

Supplementary file, clear version

## Data Availability

The datasets used in the current study as well as the codes used for meta-analysis are available from the corresponding author on reasonable request.

## References

[1] Hyman, D. M., Taylor, B. S. & Baselga, J. Implementing genome-driven oncology. *Cell* **168**, 584–599 (2017). 28187282 10.1016/j.cell.2016.12.015 PMC5463457

[2] Tsimberidou, A. M. et al. Molecular tumour boards—current and future considerations for precision oncology. *Nat. Rev. Clin. Oncol.* **20**, 843–863 (2023). 37845306 10.1038/s41571-023-00824-4

[3] Rosenquist, R., Frohling, S. & Stamatopoulos, K. Precision medicine in cancer: a paradigm shift. *Semin. Cancer Biol.* **84**, 1–2 (2022). 35597437 10.1016/j.semcancer.2022.05.008

[4] Hoadley, K. A. et al. Multiplatform analysis of 12 cancer types reveals molecular classification within and across tissues of origin. *Cell* **158**, 929–944 (2014). 25109877 10.1016/j.cell.2014.06.049 PMC4152462

[5] Bitzer, M. et al. Next-generation sequencing of advanced GI tumors reveals individual treatment options. *JCO Precis. Oncol.* **4**, PO.19.00359 (2020). 10.1200/PO.19.00359 PMC7446530 32923905

[6] Hoefflin, R. et al. Transitioning the molecular tumor board from proof of concept to clinical routine: a German single-center analysis. *Cancers (Basel)* **13**, 1151 (2021). 10.3390/cancers13051151 PMC7962829 33800365

[7] Luchini, C., Lawlor, R. T., Milella, M. & Scarpa, A. Molecular tumor boards in clinical practice. *Trends Cancer* **6**, 738–744 (2020). 32517959 10.1016/j.trecan.2020.05.008

[8] Stenzinger, A. et al. Trailblazing precision medicine in Europe: a joint view by genomic medicine Sweden and the centers for personalized medicine, ZPM, in Germany. *Semin. Cancer Biol.* **84**, 242–254 (2022). 34033893 10.1016/j.semcancer.2021.05.026

[9] Illert, A. L. et al. The German network for personalized medicine to enhance patient care and translational research. *Nat. Med.* **29**, 1298–1301 (2023). 37280276 10.1038/s41591-023-02354-z

[10] Fioretos, T. et al. Implementing precision medicine in a regionally organized healthcare system in Sweden. *Nat. Med.* **28**, 1980–1982 (2022). 36123428 10.1038/s41591-022-01963-4

[11] Tasken, K. et al. A national precision cancer medicine implementation initiative for Norway. *Nat. Med.* **28**, 885–887 (2022). 35513529 10.1038/s41591-022-01777-4

[12] Tamborero, D. et al. The molecular tumor board portal supports clinical decisions and automated reporting for precision oncology. *Nat. Cancer* **3**, 251–261 (2022). 35221333 10.1038/s43018-022-00332-x PMC8882467

[13] Larson, K. L. et al. Clinical outcomes of molecular tumor boards: a systematic review. *JCO Precis. Oncol.* **5**, PO.20.00495 (2021). 10.1200/PO.20.00495 PMC8277300 34632252

[14] Eisenhauer, E. A. et al. New response evaluation criteria in solid tumours: revised RECIST guideline (version 1.1). *Eur. J. Cancer* **45**, 228–247 (2009). 19097774 10.1016/j.ejca.2008.10.026

[15] Mateo, J. et al. A framework to rank genomic alterations as targets for cancer precision medicine: the ESMO scale for clinical actionability of molecular targets (ESCAT). *Ann. Oncol.* 29, 1895–1902 (2018). 10.1093/annonc/mdy263 PMC6158764 30137196

[16] Leichsenring, J. et al. Variant classification in precision oncology. *Int. J. Cancer* **145**, 2996–3010 (2019). 31008532 10.1002/ijc.32358

[17] Von Hoff, D. D. et al. Pilot study using molecular profiling of patients’ tumors to find potential targets and select treatments for their refractory cancers. *J. Clin. Oncol.* **28**, 4877–4883 (2010). 20921468 10.1200/JCO.2009.26.5983

[18] Rodon, J. et al. Genomic and transcriptomic profiling expands precision cancer medicine: the WINTHER trial. *Nat. Med.* **25**, 751–758 (2019). 31011205 10.1038/s41591-019-0424-4 PMC6599610

[19] Sicklick, J. K. et al. Molecular profiling of cancer patients enables personalized combination therapy: the I-PREDICT study. *Nat. Med.* **25**, 744–750 (2019). 31011206 10.1038/s41591-019-0407-5 PMC6553618

[20] Gambardella, V. et al. Molecular profiling of advanced solid tumours. The impact of experimental molecular-matched therapies on cancer patient outcomes in early-phase trials: the MAST study. *Br. J. Cancer* **125**, 1261–1269 (2021). 34493820 10.1038/s41416-021-01502-x PMC8548537

[21] Repetto, M. et al. Molecular tumour board at European institute of oncology: report of the first three year activity of an Italian precision oncology experience. *Eur. J. Cancer* **183**, 79–89 (2023). 36801623 10.1016/j.ejca.2023.01.019

[22] Reda, M. et al. Implementation and use of whole exome sequencing for metastatic solid cancer. *EBioMedicine* **51**, 102624 (2020). 31923800 10.1016/j.ebiom.2019.102624 PMC7000332

[23] Bertucci, F. et al. Prospective high-throughput genome profiling of advanced cancers: results of the PERMED-01 clinical trial. *Genome Med.* **13**, 87 (2021). 34006291 10.1186/s13073-021-00897-9 PMC8132379

[24] Liu, A. et al. Molecular tumor boards: the next step towards precision therapy in cancer care. *Hematol Rep* **15**, 244–255 (2023). 37092519 10.3390/hematolrep15020025 PMC10123678

[25] Boileve, A. et al. Molecular profiling and target actionability for precision medicine in neuroendocrine neoplasms: real-world data. *Eur. J. Cancer* **186**, 122–132 (2023). 37062210 10.1016/j.ejca.2023.03.024

[26] Debien, V. et al. Molecular analysis for refractory rare cancers: sequencing battle continues - learnings for the MOSCATO-01 study. *Crit. Rev. Oncol. Hematol.* **181**, 103888 (2023). 36460264 10.1016/j.critrevonc.2022.103888

[27] El Helali, A. et al. The impact of the multi-disciplinary molecular tumour board and integrative next generation sequencing on clinical outcomes in advanced solid tumours. *Lancet Reg. Health West Pac.* **36**, 100775 (2023). 37547050 10.1016/j.lanwpc.2023.100775 PMC10398587

[28] Fukada, I. et al. Prognostic impact of cancer genomic profile testing for advanced or metastatic solid tumors in clinical practice. *Cancer Sci.* **114**, 4632–4642 (2023). 37858313 10.1111/cas.15993 PMC10728004

[29] Giacomini, P. et al. The molecular tumor board of the regina elena national cancer institute: from accrual to treatment in real-world. *J. Transl. Med.* **21**, 725 (2023). 37845764 10.1186/s12967-023-04595-5 PMC10577953

[30] Huang, B. et al. Molecular tumor board review and improved overall survival in non-small-cell lung cancer. *JCO Precis. Oncol.* **5**, PO.21.00210 (2021). 10.1200/PO.21.00210 PMC8492377 34622117

[31] Kato, S. et al. Real-world data from a molecular tumor board demonstrates improved outcomes with a precision N-of-One strategy. *Nat. Commun.* **11**, 4965 (2020). 33009371 10.1038/s41467-020-18613-3 PMC7532150

[32] Lamping, M. et al. Support of a molecular tumour board by an evidence-based decision management system for precision oncology. *Eur. J. Cancer* **127**, 41–51 (2020). 31982633 10.1016/j.ejca.2019.12.017

[33] Louie, B. H. et al. Precision medicine-based therapies in advanced colorectal cancer: the University of California San Diego molecular tumor board experience. *Mol. Oncol.* **16**, 2575–2584 (2022). 35238467 10.1002/1878-0261.13202 PMC9251876

[34] Louie, B. H. et al. Pan-cancer molecular tumor board experience with biomarker-driven precision immunotherapy. *NPJ Precis. Oncol.* **6**, 67 (2022). 36138116 10.1038/s41698-022-00309-0 PMC9500013

[35] Mosteiro, M. et al. Molecular profiling and feasibility using a comprehensive hybrid capture panel on a consecutive series of non-small-cell lung cancer patients from a single centre. *ESMO Open* **8**, 102197 (2023). 38070435 10.1016/j.esmoop.2023.102197 PMC10774954

[36] Pinet, S. et al. Clinical management of molecular alterations identified by high throughput sequencing in patients with advanced solid tumors in treatment failure: real-world data from a French hospital. *Front Oncol.* **13**, 1104659 (2023). 36923436 10.3389/fonc.2023.1104659 PMC10009270

[37] Scheiter, A. et al. Critical evaluation of molecular tumour board outcomes following 2 years of clinical practice in a comprehensive cancer centre. *Br. J. Cancer* **128**, 1134–1147 (2023). 36572733 10.1038/s41416-022-02120-x PMC10006213

[38] Tarawneh, T. S. et al. Combined focused next-generation sequencing assays to guide precision oncology in solid tumors: a retrospective analysis from an institutional molecular tumor board. *Cancers (Basel)* **14**, 4430 (2022). 10.3390/cancers14184430 PMC9496918 36139590

[39] Nakamura, Y. et al. Targeted therapy guided by circulating tumor DNA analysis in advanced gastrointestinal tumors. *Nat. Med.* **14**, 165–175 (2024). 10.1038/s41591-024-03244-8 PMC11750700 39284955

[40] Mishra-Kalyani, P. S. et al. External control arms in oncology: current use and future directions. *Ann. Oncol.* **33**, 376–383 (2022). 35026413 10.1016/j.annonc.2021.12.015

[41] Subbiah, V. The next generation of evidence-based medicine. *Nat. Med.* **29**, 49–58 (2023). 36646803 10.1038/s41591-022-02160-z

[42] Joanna Briggs Institute. *Critical Appraisal Tools.* https://jbi.global/critical-appraisal-tools (2024).

[43] StataCorp L. L. C. *Stata Statistical Software: v. 15.1* https://www.stata.com/ (2017).

[44] Hlevnjak, M. et al. CATCH: A prospective precision oncology trial in metastatic breast cancer. *JCO. Precis. Oncol.* **5**, PO.20.00248 (2021). 10.1200/PO.20.00248 PMC8140780 34036222

[45] Horak, P. et al. Comprehensive genomic and transcriptomic analysis for guiding therapeutic decisions in patients with rare cancers. *Cancer Discov.* **11**, 2780–2795 (2021). 34112699 10.1158/2159-8290.CD-21-0126

[46] Koopman, B. et al. Relevance and effectiveness of molecular tumor board recommendations for patients with non-small-cell lung cancer with rare or complex mutational profiles. *JCO Precis. Oncol.* **4**, 393–410 (2020). 35050740 10.1200/PO.20.00008

[47] Martin-Romano, P. et al. Implementing the European society for medical oncology scale for clinical actionability of molecular targets in a comprehensive profiling program: impact on precision medicine oncology. *JCO Precis. Oncol.* **6**, e2100484 (2022). 36315916 10.1200/PO.21.00484

[48] Miller, R. W. et al. Molecular tumor board-assisted care in an advanced cancer population: results of a phase II clinical trial. *JCO Precis. Oncol.* **6**, e2100524 (2022). 36103643 10.1200/PO.21.00524 PMC9489195

[49] Pleasance, E. et al. Whole-genome and transcriptome analysis enhances precision cancer treatment options. *Ann. Oncol.* **33**, 939–949 (2022). 35691590 10.1016/j.annonc.2022.05.522

[50] Sultova, E. et al. NGS-guided precision oncology in metastatic breast and gynecological cancer: first experiences at the CCC Munich LMU. *Arch. Gynecol. Obstet.* **303**, 1331–1345 (2021). 33277683 10.1007/s00404-020-05881-z PMC8053190

[51] Sultova, E. et al. Implementation of precision oncology for patients with metastatic breast cancer in an interdisciplinary MTB setting. *Diagnostics (Basel)* **11**, 733 (2021). 10.3390/diagnostics11040733 PMC8074310 33924134

[52] Cobain, E. F. et al. Assessment of clinical benefit of integrative genomic profiling in advanced solid tumors. *JAMA Oncol.* **7**, 525–533 (2021). 33630025 10.1001/jamaoncol.2020.7987 PMC7907987

[53] Bayle, A. et al. Clinical utility of circulating tumor DNA sequencing with a large panel: a national center for precision medicine (PRISM) study. *Ann. Oncol.* **34**, 389–396 (2023). 36709039 10.1016/j.annonc.2023.01.008

[54] Zhang, D. et al. A retrospective analysis of biliary tract cancer patients presented to the molecular tumor board at the comprehensive cancer center Munich. *Target Oncol.* **18**, 767–776 (2023). 37594677 10.1007/s11523-023-00985-3 PMC10517894

[55] Ladekarl, M. et al. Feasibility and early clinical impact of precision medicine for late-stage cancer patients in a regional public academic hospital. *Acta. Oncol.* **62**, 261–271 (2023). 36905645 10.1080/0284186X.2023.2185542

[56] Renovanz, M. et al. Clinical outcome of biomarker-guided therapies in adult patients with tumors of the nervous system. *Neurooncol. Adv.* **5**, vdad012 (2023). 36915613 10.1093/noajnl/vdad012 PMC10007909

[57] Blobner, J. et al. Significance of molecular diagnostics for therapeutic decision-making in recurrent glioma. *Neurooncol. Adv*. **5**, vdad060 (2023). 37287694 10.1093/noajnl/vdad060 PMC10243988

