# Supplementary material for: Systematic review and meta-analysis of molecular tumor board data on clinical effectiveness and evaluation gaps
Source: NPJ Precis Oncol. 2025 Apr 2;9:96. doi: 10.1038/s41698-025-00865-1 (PMC11965449; doi:10.1038/s41698-025-00865-1)
Supplement: Supplementary file 1 — Supplementary file, clear version [file 41698_2025_865_MOESM1_ESM.pdf]

Supplementary material for

Systematic Review and Meta-Analysis of Molecular Tumor Board Data on Clinical Effectiveness and Evaluation Gaps

Beryl Primrose Gladstone[1,2], Janina Beha[3], Arisa Hakariya[1], Pavlos Missios[1,3,4], Nisar P. Malek[1,3,4], Michael Bitzer[1,3,4]

[1]Department of Internal Medicine I, Eberhard-Karls University Tuebingen, Germany

[2]DZIF-Clinical research unit, Infectious diseases, Internal Medicine I, Eberhard-Karls University, Tuebingen, Germany

[3]Center for Personalized Medicine, Eberhard-Karls University, Tübingen, Germany

[4]M3-Research Center for Malignome, Metabolome and Microbiome, Eberhard-Karls University, Tuebingen, Germany.

Supplementary Table 1: Descriptive characteristics of the included studies (N=34)

Study characteristics	Number of studies (%) N=34
**Study design**	
Clinical trial	5 (14.7%)
Prospective cohort study	10 (29.4%)
Retrospective cohort study	19 (55.8%)
**Country**	
Germany	12 (35.3%)
France	7 (20.6%)
USA	6 (17.7%)
Spain	2 (5.9%)
Italy	2 (5.9%)
Canada	1 (2.9%)
Netherlands	1 (2.9%)
Japan	1 (2.9%)
China	1 (2.9%)
Denmark	1 (2.9%)
**Cancer type**	
All advanced/metastatic/refractory cancer	21 (61.8%)
Non-small cell lung cancer	3 (8.8%)
Advanced breast / gynaecological cancer	3 (8.8%)
Gastro-intestinaI cancer	3 (8.8%)
Nervous system cancer	2 (5.8%)
Neuroendocrine cancer	1 (2.9%)
Advanced rare cancer	1 (2.9%)
**Duration of data collection**	
1 to 2 years	6 (17.6%)
3 to 4 years	14 (41.4%)
5 or more years	14 (41.4%)
**RECIST criteria followed**	
No	14 (41.2%)
Yes	20 (58.8%)
**Definition for stable disease** (if reported) **(N=18)**	
At least 6 weeks	1 (5.6%)
At least 8 weeks	10 (55.6%)
At least 3 months	3 (16.7%)
At least 6 months	4 (22.2%)
**Actionability scale used**	
None reported	15 (44%)
ESCAT[1]	10 (29%)
ESCAT and NCT/DKTK[2] evidence level	3 (8.8%)
NCT/DKTK evidence level	1 (2.9%)
OncoKB[3]	1 (2.9%)
University of Kentucky grading of evidence[4]	1 (2.9%)
Modified evidence levels[5]	1 (2.9%)
Evidence level according to Meric-Bernstam et al.[6]	1 (2.9%)
ASCO, AMP, CAP consensus[7]	1 (2.9%)
**Comparison group to assess the impact of MTB***	
MTB referred patients that did not get treated according to MTB-recommended therapy / patients getting still available standard of care therapies	15 (44.1%)

MTB referred patients who were not given any treatment	2 (5.9%)
MTB referred patients with no actionable driver / no MTB recommendation	2 (5.9%)
Patients with low study-defined matching score	2 (5.9%)
Patients who were not referred to MTB	1 (2.9%)
No comparison	15 (44.1%)
**Outcomes reported**	
Objective response rate	20 (58.8%)
Disease control rate	23 (67.7%)
Overall survival (OS)	22 (64.7%)
Progression free survival (PFS)	26 (76.5%)

*Two different comparisons were made in each of three studies.
Superscript numbers refer to the reference that defined the actionability scale.

Supplementary Figure 1: Major tumor entities presented to the MTB in the included studies (N=34). The bars indicate the patient's cancer entity grouped into major categories wherever possible. The cancer entities of all MTB referred patients were available for only 10731 patients as the distribution of the cancer entities was reported for all MTB referred patients in 23 studies and specific patient groups in 11 studies.

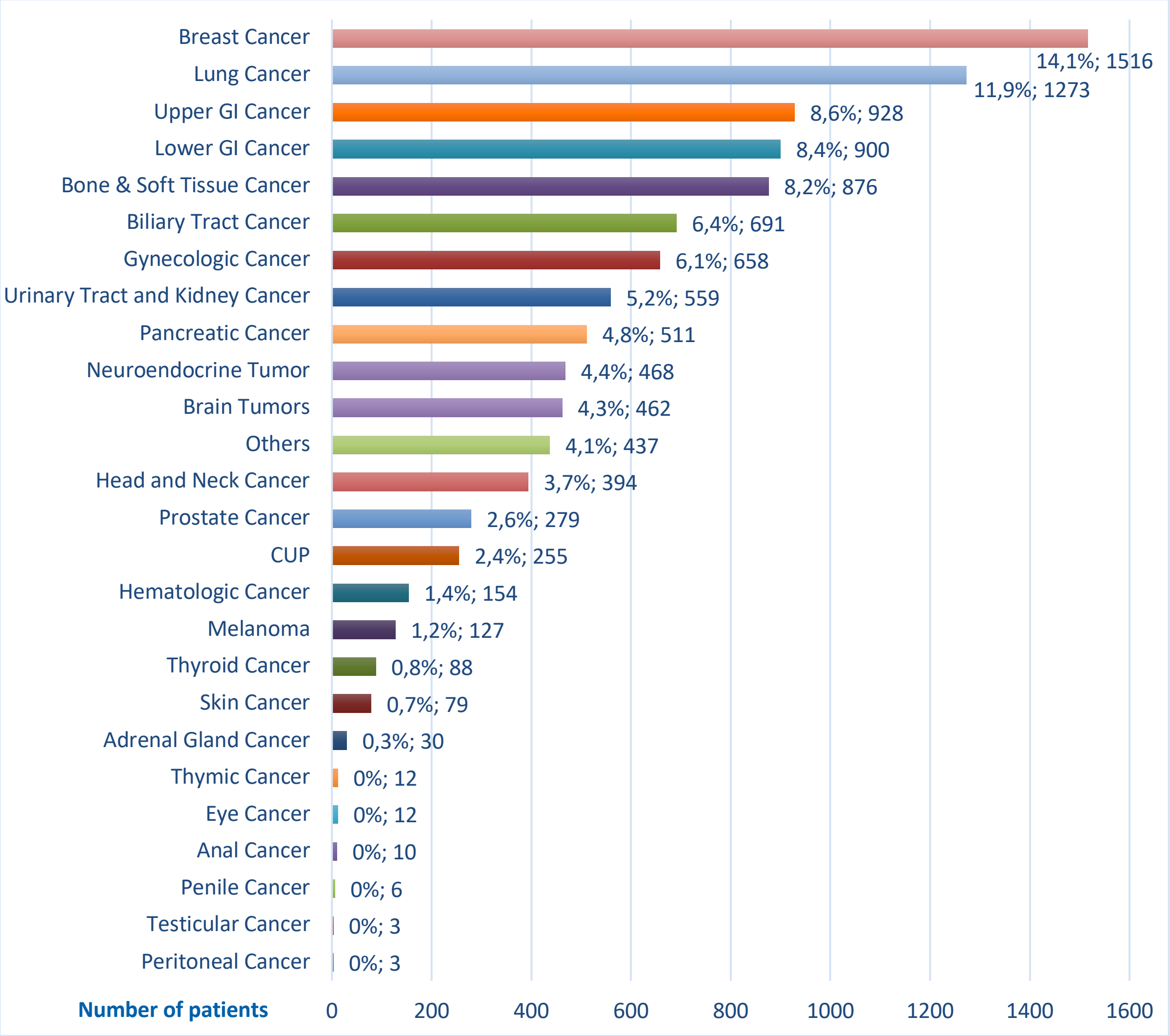

Supplementary Table 2: Definitions of outcomes used in single studies

Author & year	Outcome	Definition in the publication
Cobain and Wu, 2021[5]	CBRtt_dur (Clinical benefit based on treatment duration)	Proportion of patients receiving sequencing-directed therapy for 6 months or longer
Cobain and Wu, 2021[5]	EXR (Exceptional responder rate)	Exceptional responders received SDT for a duration of 12 months or longer
Zhang, 2023[8]	mDoR (Median duration of response)	Not provided
Renovanz, Kurz, and Rieger, 2023[9]	mDoCB (Median duration of clinical benefit)	Not provided

Supplementary Table 3: Actionability scales and its association with outcomes reported in the MTB studies:

Study number	First author	Actionability scale(s) (ACTS)	Specific outcome studied for ACTS	Association of outcome to ACTS Level*	Details of ACTS_outcome_results (as reported by the study authors)
6	Horak, Heining, Kreutzfeldt, and Hutter [10]	NCT/DKTK Evidence Levels, ESCAT	$pPFSR_{\geq 1.3}$	associated	**Proportion of pPFSR >1.3** highest for highest level of evidence (proportions for the different scales are given in brackets):<br>NCT/DKTK evidence levels m1A–C (55%–72%), m2A–C (21%–36%), and m3–4 (34%–36%).<br>ESCAT tiers I–II (25%–83%) and IIIA–B (34%–38%).
13	Martin-Romano[11]	ESCAT	ORR, PFS, OS	ORR and PFS - associated<br><br>OS - not associated	**Best objective response**: ESCAT tier I (PR/SD: 70%; PD: 30%), II (PR/SD: 60%; PD: 40%), tier III (PR/SD: 34%; PD: 63%) and IV (PR/SD: 27%; PD: 73%).<br>**Median PFS**: ESCAT I - 6.5 months (95% CI, 4.2 to 8.9), II - 3 months (95% CI, 1 to [NA]), III - 3 months (95% CI, 2.2 to 3.8), and IV - 4 months (95% CI, 2.8 to 6.3).<br>**Median OS**: ESCAT I - 8.4 months (95% CI, 6.6 to 10), II - 8.8 months (95% CI, 1.6 to NA), III - 6.8 months (95% CI, 4.5 to 12.5), and IV - 6 months (95% CI, 4.9 to 10.9)
14	Miller and Hutchcraft[4]	University of Kentucky Grading of Evidence	$pPFSR_{\geq 1.3}$	not associated	Level 1 evidence: a Kaplan-Meier estimate of **P(GMI ≥ 1.3)** was 0.61 (95% CI, 0.39 to 0.77), and the median PFS ratio was 2.053 (95% CI, 0.85 to 2.88) for patients treated.<br>Level 2 or 3 evidence: a Kaplan-Meier estimate of **P(GMI ≥ 1.3)** was 0.57 (95% CI, 0.34 to 0.74), and the median PFS ratio was 1.551 (95% CI, 0.54 to 2.56) for patients treated.<br><br>There was no difference between these groups (log-rank test; P = .54), suggesting that benefit of MTB-directed therapy was similar regardless of the evidence level.
24	Giacomini[12]	OncoKB	ORR	not associated	The authors were unable to establish significant correlations between the OncoKB level (1/2 or 3A/B) and either objective response rate or TTP of MTB-assigned treatment.
25	Repetto and Crimini[13]	ESCAT	PFS, OS	associated - OS, PFS- Not associated	Significant difference was detected in **OS** but not in **PFS** considering ESCAT tiers of macthed therapy indication (p = 0.03 and p = 0.25, respectively).<br>Patients who received therapy in the ESCAT tier I had better **OS** than patients not receiving targeted treatment (p = 0.001) and a numerically but not statistically significant advantage in **PFS** (p = 0.49).<br><br>No difference in **OS and PFS** was observed for ESCAT III patients versus non-matched treatment and for all the patients receiving matched treatment excluding ESCAT I patients versus non-matched therapy.

31	Fukada[14]	ESCAT	OS	Not associated	The median survival time of patients who received genomically matched therapy with ESCAT I/II (N = 9) was 19.8 months (95% CI, 3.2–36.4), while those of patients who did not receive genomically matched therapy (N = 668) was 14.2 months (95% CI, 12.4–16.0).<br><br>No significant differences were noted in OS between patients who received genomically matched therapy with ESCAT I/II and those who did not (p = 0.653).
32	Scheiter [15]	NCT/DKTK	CBR	Associated	Among the patients experiencing clinical benefit from MTB therapies, 63.2% had evidence levels of m1A (12/19), 15.8% had m1C evidence (3/19), and 5.3% had m1B, m2A, m2C and m3 evidence (1/19 each).<br><br>This distribution differs among patients who did not experience clinical benefit from MTB-recommended therapies, where only 52.6% of patients (10/19) had m1 evidence, while 47.4% (9/19) had evidence level of m2 or below.

Note: *Associated refers to significant association of the actionability scale to study defined clinical outcome. PFS, progression free survival; $pPFSR_{\geq 1.3}$, the percentage of the patients with a PFS2/PFS1 ratio $\geqq$1.3; PR, partial response rate; CR, complete response rate; DC, disease control; OS, overall survival; ORR, objective response rate; DCR, disease control rate (CR+PR+SD); CBR, clinical benefit rate; DoR, duration of response; DoCB, duration of clinical benefit; ACTS, actionability scales; GMI, Growth modulation index; P(GMI ≥ 1.3) corresponds to $pPFSR_{\geq 1.3}$

Supplementary Table 4: Definitions of “matched therapy/matching/matching score” among MTB based data

First Author & year	Term "Matching"	Definition
Bertucci, 2021[16]	Matched therapy	Defined as “matched” when its prescription was based upon an AGA (actionable genetic alteration) identified
Kato and Kim, 2020[17]	Matching score	Matching score evaluated the number of pathogenic alterations targeted by drugs given divided by total number of pathogenic alterations
Kato, 2022[18]	Matching score	Matching Score was roughly defined as the number of alterations (not counting variants of unknown significance, VUS) targeted by administered drugs, divided by the total number of pathogenic alterations (not counting VUSs) discerned (no differentiation between potential driver versus passenger alterations).
Lamping and Benary, 2020[19]	Matched cohort	Patients with a treatment option recommendation at the time of data cut-off had received treatment as recommended by the MTB are included in the matched cohort. The patients who received treatments other than recommended by the MTB are included in the unmatched treatment cohort
Louie and Kato, 2022[20]	Matched therapy	Treatment was considered “matched” if $\geq$ 1 compound in the therapy regimen targeted $\geq$ 1 aberration or pathway component aberrant in a patient’s molecular profile or a functionally active protein preferentially expressed in the tumor with an IC_{50} value in the low nmol/L range (for small molecule inhibitors) or if the aberration was the primary target for antibodies. Checkpoint blockade was considered matched if the patient had intermediate or high tumor mutation burden (TMB), positive immunohistochemistry for PDL1 or specific tumor alterations such as PDL1 amplification.
Louie and Kato, 2022[21]	Matching score	The Matching Score calculation included evaluation of all NGS characterized variants (but not variants of unknown significance [VUS]) as well as mRNA expression, protein expression, and immunotherapy biomarkers in select cases.<br><br>The Matching Score was calculated by taking the number of alterations targeted by drugs given divided by the total number of alterations. In the case of immunotherapy, scoring also considered immune biomarkers.
Boilève, 2023[22]	Molecularly matched treatment (MMT)	Classification of actionability of MA was performed based on the ESCAT classification. The median OS since metastatic diagnosis of patients who recieved an MMT at any time during their disease time course was compared to patients that did not get MMT.

Supplementary Figure 2: Risk of bias assessment of the included studies using adapted JBI critical appraisal tool (N=34).

Risk of bias

First author/ Year	D1	D2	D3	D4	D5	D6	D7	D8	D9	D10	D11	Overall
Bertucci 2021	+	+	+	+	+	+	+	+	+	+	+	+
Bitzer and Ostermann 2020	X						+	+	+	+	X	-
Gambardella 2021	+	+	+	+	+	+	+	+	+	+	+	+
Hlevnjak, Schulze, and Elgaafary 2021	X						+	+	+	+	X	-
Hoefflin 2021	+	+	+	X	X	X	+	+	+	+	X	-
Horak, Heining, Kreutzfeldt, and Hutter 2021	X						-	+	X	+	X	X
Huang 2021	+	+	+	+	+	+	X	-	X	+	+	+
Kato and Kim 2020	+	+	+	X	X	X	+	+	+	+	+	+
Koopman 2020	X						+	+	+	+	X	-
Lamping and Benary 2020	X						-	+	+	+	X	-
Louie and Kato 2022a	+	+	+	X	X	X	+	+	+	+	+	-
Louie and Kato 2022b	+	+	+	X	+	+	+	+	X	+	+	-
Martin-Romano 2022	X						+	+	X	+	X	X
Miller and Hutchcraft 2022	X						-	+	+	+	X	-
Pleasance and Bohm 2022	X						+	+	+	+	X	-
Reda 2020	+	+	+	X	X	X	+	+	+	+	X	-
Sultova 2020	X						-	+	+	+	X	-
Sultova 2021	X						+	+	+	+	X	-
Tarawneh 2022	+	+	X	X	X	X	-	+	+	+	X	-
Cobain and Wu 2021	X						+	+	+	+	X	-
Bayle and Belcaid 2023	X						+	+	X	+	+	-
Boilève 2023	+	+	+	X	X	X	+	+	+	+	+	-
Zhang 2023	X						+	+	+	+	+	+
Giacomini 2023	+	+	+	X	X	X	+	+	+	+	+	-
Repetto and Crimini 2023	+	+	+	+	+	+	+	+	+	+	+	+
Helali 2023	+	+	+	X	X	X	+	+	X	+	+	-
Debien 2023	+	+	+	X	X	X	+	+	+	+	+	-
Ladekarl 2023	X						+	+	+	+	X	-
Renovanz, Kurz, and Rieger 2023	X						+	+	+	+	X	-
Pinet and Durand 2023	+	+	+	X	+	+	+	+	+	+	+	+
Fukada 2023	+	+	+	X	X	X	+	+	+	+	+	-
Scheiter 2022	+	+	+	+	X	X	+	+	+	+	+	+
Blobner 2023	X						+	+	+	+	X	-
Mosteiro, Azuara, Villatoro, and Alay 2023	+	+	+	X	X	+	+	+	X	+	+	-

D1: Controlled?
D2: Selection bias?
D3: Exposure ascertainment?
D4: Other differences?
D5: Presence of confounders studied?
D6: Confounders adjusted for?
D7: Outcome definition?
D8: Follow-up time long enough?
D9: Follow-up data provided?
D10: Incomplete follow-up considered in analysis?
D11: Statistical analysis?

Judgement
- X High
- \- Unclear
- \+ Low
- Not applicable

Supplementary Figure 3: A summary chart of the risk of bias assessment of the included studies using adapted JBI critical appraisal tool (N=34). Green shaded area represent the proportion of studies with low risk of the specific bias; red represents high risk of specific bias; yellow represents unclear information and blue refers to the specific risk assessment is not applicable.

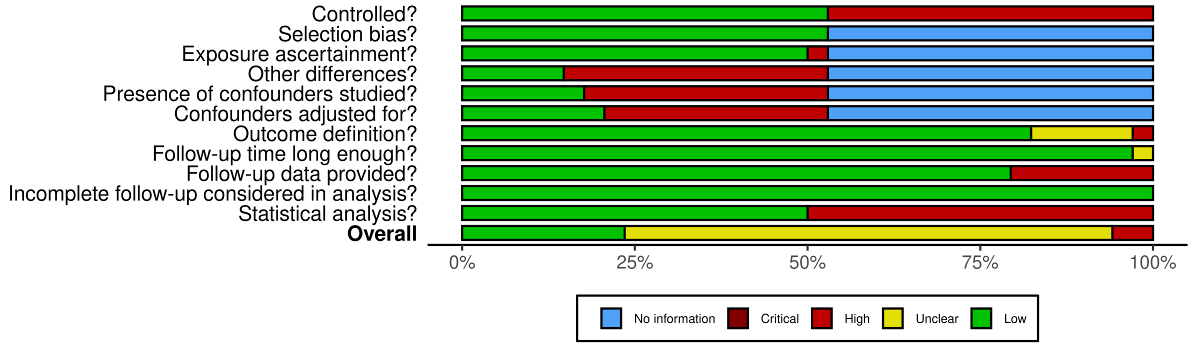

Supplementary Figure 4: Possible control groups for a comparison of MTB-guided treatments. Dashed lines indicate groups that might be used for the comparison with MTB-guided treatments.

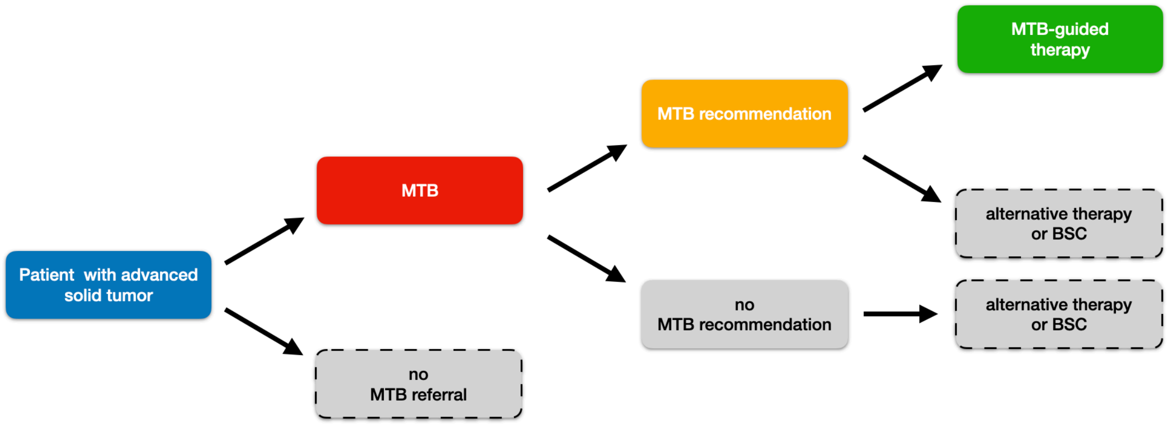

Supplementary References

1 Mateo, J. *et al.* A framework to rank genomic alterations as targets for cancer precision medicine: the ESMO Scale for Clinical Actionability of molecular Targets (ESCAT). *Ann Oncol* **29**, 1895-1902 (2018). https://doi.org/10.1093/annonc/mdy263

2 Leichsenring, J. *et al.* Variant classification in precision oncology. *Int J Cancer* **145**, 2996-3010 (2019). https://doi.org/10.1002/ijc.32358

3 Chakravarty, D. *et al.* OncoKB: A Precision Oncology Knowledge Base. *JCO Precis Oncol* **2017** (2017). https://doi.org/10.1200/PO.17.00011

4 Miller, R. W. *et al.* Molecular Tumor Board-Assisted Care in an Advanced Cancer Population: Results of a Phase II Clinical Trial. *JCO Precis Oncol* **6**, e2100524 (2022). https://doi.org/10.1200/PO.21.00524

5 Cobain, E. F. *et al.* Assessment of Clinical Benefit of Integrative Genomic Profiling in Advanced Solid Tumors. *JAMA Oncol* **7**, 525-533 (2021). https://doi.org/10.1001/jamaoncol.2020.7987

6 Meric-Bernstam, F. *et al.* A decision support framework for genomically informed investigational cancer therapy. *J Natl Cancer Inst* **107** (2015). https://doi.org/10.1093/jnci/djv098

7 Li, M. M. *et al.* Standards and Guidelines for the Interpretation and Reporting of Sequence Variants in Cancer: A Joint Consensus Recommendation of the Association for Molecular Pathology, American Society of Clinical Oncology, and College of American Pathologists. *J Mol Diagn* **19**, 4-23 (2017). https://doi.org/10.1016/j.jmoldx.2016.10.002

8 Zhang, D. *et al.* A Retrospective Analysis of Biliary Tract Cancer Patients Presented to the Molecular Tumor Board at the Comprehensive Cancer Center Munich. *Target Oncol* **18**, 767-776 (2023). https://doi.org/10.1007/s11523-023-00985-3

9 Renovanz, M. *et al.* Clinical outcome of biomarker-guided therapies in adult patients with tumors of the nervous system. *Neurooncol Adv* **5**, vdad012 (2023). https://doi.org/10.1093/noajnl/vdad012

10 Horak, P. *et al.* Comprehensive Genomic and Transcriptomic Analysis for Guiding Therapeutic Decisions in Patients with Rare Cancers. *Cancer Discov* **11**, 2780-2795 (2021). https://doi.org/10.1158/2159-8290.CD-21-0126

11 Martin-Romano, P. *et al.* Implementing the European Society for Medical Oncology Scale for Clinical Actionability of Molecular Targets in a Comprehensive Profiling Program: Impact on Precision Medicine Oncology. *JCO Precis Oncol* **6**, e2100484 (2022). https://doi.org/10.1200/PO.21.00484

12 Giacomini, P. *et al.* The Molecular Tumor Board of the Regina Elena National Cancer Institute: from accrual to treatment in real-world. *J Transl Med* **21**, 725 (2023). https://doi.org/10.1186/s12967-023-04595-5

13 Repetto, M. *et al.* Molecular tumour board at European Institute of Oncology: Report of the first three year activity of an Italian precision oncology experience. *Eur J Cancer* **183**, 79-89 (2023). https://doi.org/10.1016/j.ejca.2023.01.019

14 Fukada, I. *et al.* Prognostic impact of cancer genomic profile testing for advanced or metastatic solid tumors in clinical practice. *Cancer Sci* **114**, 4632-4642 (2023). https://doi.org/10.1111/cas.15993

15 Scheiter, A. *et al.* Critical evaluation of molecular tumour board outcomes following 2 years of clinical practice in a Comprehensive Cancer Centre. *Br J Cancer* **128**, 1134-1147 (2023). https://doi.org/10.1038/s41416-022-02120-x

16 Bertucci, F. *et al.* Prospective high-throughput genome profiling of advanced cancers: results of the PERMED-01 clinical trial. *Genome Med* **13**, 87 (2021). https://doi.org/10.1186/s13073-021-00897-9

17 Kato, S. *et al.* Real-world data from a molecular tumor board demonstrates improved outcomes with a precision N-of-One strategy. *Nat Commun* **11**, 4965 (2020). https://doi.org/10.1038/s41467-020-18613-3
18 Kato, S. *et al.* Multi-omic analysis in carcinoma of unknown primary (CUP): therapeutic impact of knowing the unknown. *Mol Oncol* **18**, 956-968 (2024). https://doi.org/10.1002/1878-0261.13293
19 Lamping, M. *et al.* Support of a molecular tumour board by an evidence-based decision management system for precision oncology. *Eur J Cancer* **127**, 41-51 (2020). https://doi.org/10.1016/j.ejca.2019.12.017
20 Louie, B. H. *et al.* Precision medicine-based therapies in advanced colorectal cancer: The University of California San Diego Molecular Tumor Board experience. *Mol Oncol* **16**, 2575-2584 (2022). https://doi.org/10.1002/1878-0261.13202
21 Louie, B. H. *et al.* Pan-cancer molecular tumor board experience with biomarker-driven precision immunotherapy. *NPJ Precis Oncol* **6**, 67 (2022). https://doi.org/10.1038/s41698-022-00309-0
22 Boileve, A. *et al.* Molecular profiling and target actionability for precision medicine in neuroendocrine neoplasms: real-world data. *Eur J Cancer* **186**, 122-132 (2023). https://doi.org/10.1016/j.ejca.2023.03.024

The references of the manuscripts that have been included in the metanalysis are all given in the main part of the manuscript.
